# Predictive brain activity related to auditory information is associated with performance in speech comprehension tasks in noisy environments

**DOI:** 10.3389/fnhum.2024.1479810

**Published:** 2024-10-30

**Authors:** Kazuhiro Okamoto, Kengo Hoyano, Yoshitomo Saiki, Tomomi Nomura, Keisuke Irie, Naoya Obama, Narihiro Kodama, Yasutaka Kobayashi

**Affiliations:** ^1^Department of Rehabilitation, Faculty of Health Science, Fukui Health Science University, Fukui, Japan; ^2^Cognitive Motor Neuroscience, Department of Human Health Sciences, Graduate School of Medicine, Kyoto University, Kyoto, Japan; ^3^Department of Speech and Language Therapy, Faculty of Health Rehabilitation, Kawasaki University of Medical Welfare, Kurashiki, Japan

**Keywords:** auditory processing, electroencephalography, stimulus-preceding negativity, predictive coding, speech reception

## Abstract

**Introduction:**

Understanding speech in noisy environments is challenging even for individuals with normal hearing, and it poses a significant challenge for those with hearing impairments or listening difficulties. There are limitations associated with the current methods of evaluating speech comprehension in such environments, especially in individuals with peripheral hearing impairments. According to the predictive coding model, speech comprehension is an active inference process that integrates sensory information through the interaction of bottom-up and top-down processing. Therefore, in this study, we aimed to examine the role of prediction in speech comprehension using an electrophysiological marker of anticipation: stimulus-preceding negativity (SPN).

**Methods:**

We measured SPN amplitude in young adults with normal hearing during a time-estimation task with auditory feedback under both quiet and noisy conditions.

**Results:**

The results showed that SPN amplitude significantly increased in noisy environments. Moreover, individual differences in SPN amplitude correlated with performance in a speech-in-noise test.

**Discussion:**

The increase in SPN amplitude was interpreted as reflecting the increased requirement for attentional resources for accurate prediction of speech information. These findings suggest that SPN could serve as a noninvasive neural marker for assessing individual differences in top-down processing involved in speech comprehension in noisy environments.

## 1 Introduction

Speech comprehension in noisy environments is challenging even for individuals with normal hearing and cognitive abilities (Cherry and Wiley, 1967), but it is even more difficult for those with listening difficulties (LiD; Petley et al., 2021; Parthasarathy et al., 2020). Some individuals with LiD have normal audiograms but find it extremely hard to understand speech in noisy situations. They also struggle with following complex verbal instructions and understanding rapid or degraded speech (Jerger and Musiek, 2000). Some of the children and adults who face these issues are diagnosed with auditory processing disorder (American Speech-Language-Hearing Association, 1996).

Speech comprehension relies on the integration of acoustic feature processing in the auditory pathway (bottom-up processing; Hardcastle et al., 2012) and linguistic feature processing in the speech and language pathways (top-down processing; Davis and Johnsrude, 2007). The top-down processing can be viewed as an inferential process that uses context information at different levels, such as syntax, semantics, and phonemes, to resolve ambiguities in the speech signal (Obleser, 2014). This process also involves memory and attention (Pichora-Fuller et al., 2016; Rönnberg et al., 2019), which play crucial roles. These two pathways are not mutually exclusive: bottom-up processing can influence top-down processing and vice versa (Lesicko and Llano, 2017; Morlet et al., 2019). It is plausible that many individuals with LiD who have normal audiograms have issues specifically affecting the top-down processing (Moore et al., 2010). Evaluating individual differences in neural activity during the top-down processing of speech comprehension, specifically inference processes, may be crucial for understanding the problems faced by individuals with LiD who have normal audiograms and for selecting appropriate treatment strategies.

For evaluating speech comprehension in individuals with LiD, it is common to use not only hearing tests but also behavioral auditory processing tests, such as speech-in-noise perception assessments and dichotic listening tests. However, even on following clinical guidelines, these tests can yield highly variable results (Jutras et al., 2007; Wilson and Arnott, 2013). Additionally, it is impossible to completely rule out the influence of peripheral hearing impairments, such as synaptic dysfunction between inner hair cells and type I auditory nerve fibers (Kujawa and Liberman, 2019), subclinical endolymphatic hydrops (Yoshida et al., 2023), and reduced extended high-frequency hearing (Motlagh Zadeh et al., 2019; Hunter et al., 2020), which are undetectable by audiograms and influence bottom-up processing. Neurophysiological evaluations using auditory brainstem responses (Ankmnal-Veeranna et al., 2019; Hunter et al., 2023) and event-related potentials, such as P300 (Morlet et al., 2019; Petley et al., 2024), have been reported. Although these measures provide valuable insights, they primarily examine neural activity following bottom-up processing. Therefore, similar to the behavioral auditory processing tests, these tests are limited by their inability to exclude the effects of peripheral hearing impairments that are undetectable with audiograms (Dillon and Cameron, 2021).

We propose that stimulus-preceding negativity (SPN; Brunia, 1988; Brunia and van Boxtel, 2004), an electrophysiological marker of prediction, could serve as a useful biomarker for top-down processes involved in speech comprehension. The neural dynamics underlying prediction have been studied using electroencephalogram (EEG) in an S1-S2 paradigm, where a cue stimulus (S1) is followed by a target stimulus (S2). During tasks requiring participants to respond to S2 with a motor action, brain potentials gradually shift in a negative direction between S1 and S2; this shift is known as contingent negative variation (CNV; Mento, 2013; Ruiz-Martínez et al., 2022). This phenomenon has been observed across various sensory modalities, including auditory function. Even when no motor response to S2 is required, a similar negative shift in brain potential has been reported based on mental anticipation of S2, termed SPN. SPN is characterized by a negative shift in brain potential observed prior to the arrival of sensory input, indicating that the brain is preparing for the incoming stimulus. It emerges between the cue and feedback without necessitating a direct motor response. Therefore, the SPN can be considered a predictive neural activity that is not influenced by bottom-up sensory processing or the preparation of motor responses (Brunia and Damen, 1988). Changes in the amplitude of SPN are most pronounced when individuals anticipate significant events, such as feedback on performance or decision-making outcomes (Zhang et al., 2017; Li et al., 2018; Walentowska et al., 2018).

The importance of prediction in perceptual processes has been explained within the framework of predictive coding (Friston et al., 2006; Friston, 2010). Additionally, the relationship between predictive coding theory and CNV modality, including SPN, has been further demonstrated through approaches using mathematical inference models (Bennett et al., 2015; Gómez et al., 2019). This theory posits that the brain seeks to minimize discrepancies between predictions generated by internal models and actual sensory input. This minimization is accomplished through a hierarchical system in which higher brain areas formulate predictions juxtaposed with sensory information from lower brain areas. When there is a variance between the brain’s predictions and sensory perceptions (termed “prediction error”), the brain updates its model to reduce this error. Through these prediction errors, the brain continuously learns to better anticipate future events and stimuli (Friston, 2009). This theory suggests that perception, including speech comprehension, is not merely a passive reception of sensory inputs but rather an active inferential process (Heilbron and Chait, 2018; Pezzulo et al., 2024).

Recent studies on predictive coding suggest that paying attention to stimuli enhances the gain of neurons that relay prediction errors to more advanced hierarchical inference structures (Friston, 2009; Feldman and Friston, 2010). Based on this hypothesis, auditory inputs in noisy environments could produce larger prediction errors than those in quiet settings. This is because auditory signals masked by background noise demand more attentional resources to accurately predict the original auditory information (Ono et al., 2021). When background noise reduces the clarity of the speech signal, it is expected that the level of prediction errors will also change, leading to variations in the SPN amplitude.

This study aims to clarify the relationship between SPN and performance during speech comprehension tasks in noisy environments in young adults with normal hearing. We considered the following hypotheses. (1) In noisy environments, the attentional resources required for speech comprehension increase compared to in quiet environments, which would lead to changes in SPN amplitude. (2) The change in the SPN amplitude between noisy and quiet environments would be positively correlated with performance in behavioral auditory processing tests. These hypotheses were tested using a time-estimation task with auditory feedback.

## 2 Materials and methods

### 2.1 Participants

Participants aged 18–23 years were considered eligible to minimize the influence of age-related hearing changes, and they were required to pass a preliminary evaluation involving an audiometric test to confirm normal hearing across frequencies of 125–8000 Hz. Normal hearing thresholds were defined as pure-tone thresholds of ≤ 20 dBHL (American Speech-Language-Hearing Association, 1990). An auditory continuous performance test (Schmidt et al., 2017; Schmidt et al., 2019) was conducted to ensure normal and sustained auditory attention. The final cohort of participants comprised 18 women and nine men, with an average age of 20.2 years. Hearing thresholds, measured using a three-frequency pure-tone average between 0.5 and 2 kHz in both ears, ranged from −1.9 to 11.3 dBHL, with an average of 5.6 dBHL and a standard deviation (SD) of 3.5 dBHL. The average accuracy rate in the auditory continuous performance test was 99.5%, and none of the participants exhibited signs of sustained attention impairment. All participants were confirmed to be right-handed based on the Japanese version of the simplified Edinburgh Inventory (Oldfield, 1971). No participants reported visual impairments, neurological disorders, or motor disabilities.

All the participants provided written informed consent after receiving comprehensive information about the study objectives, procedures, potential risks, and benefits. The experiment was conducted in accordance with the ethical standards of the Helsinki Declaration, and the guidelines were approved by the Regional Ethics Committee of Fukui Health Science University. This study was documented following the Strengthening the Reporting of Observational Studies in Epidemiology (STROBE) guidelines.

### 2.2 Apparatus and stimuli

**FIGURE 1:**
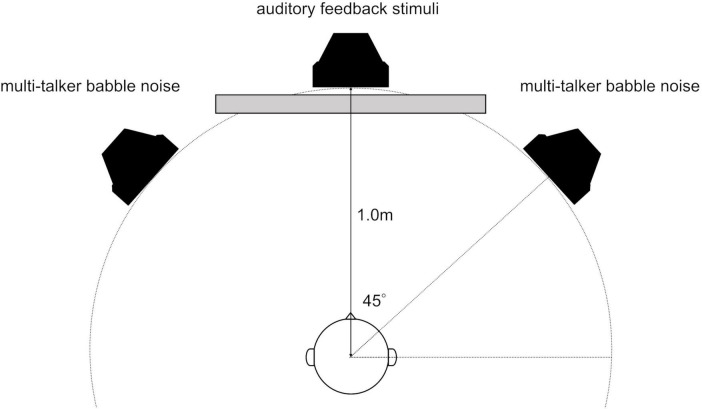
Arrangement of loudspeakers and listeners in a soundproof room. There was a 1.25 m separation between the participants and chamber wall, as well as between the loudspeakers and chamber wall.

The participants were comfortably seated in a soundproof room with loudspeakers (8330AP; GENELEC, Tokyo, Japan) placed 1 m away at the midpoint (at 0°) and to the left and right (at 45° and −45°). The central loudspeaker delivered auditory feedback for the time-estimation task, whereas the left and right loudspeakers emitted multi-talker babble noise (Figure 1). The loudspeakers were meticulously aligned with the center of the participants’ heads. There was a 1.25 m separation between the participants and chamber wall, as well as between the loudspeakers and chamber wall.

Multi-talker babble noise was generated by recording five men and five women reading a text aloud for 10 minutes. The recording was made using a condenser microphone (C480 B Combo; AKG, Wien, Austria) mounted on a boom arm and a portable multichannel recorder (DR680MK2; TASCAM, Tokyo, Japan). After digitization at a sampling rate of 48 kHz and a bit depth of 24, a low-pass filter was applied at 8 kHz (Lilly et al., 2011). To ensure that the sound image was localized in 10 directions around the participant using two loudspeakers, this noise was generated by delaying the arrival time of the noise in the left ear slightly more than that in the right ear. A sound image refers to the mental representation of the location of a sound source as perceived by an individual (Blauert, 1997). The apparent signal delay was set to achieve sound image localization angles of 82.6°, 62.3°, 48.1°, 32.1°, 16.5°, −16.5°, −32.1°, −48.1°, −62.3°, and −82.6°.

Visual stimuli, including instructions and cues, were displayed on a 14” liquid crystal display monitor situated 1 m in front of the participants. The sound pressure level of the central loudspeaker, delivering auditory feedback (target stimuli), was calibrated to 65 dB SPL. Meanwhile, the left and right loudspeakers, angled at 45° and emitting multi-talker babble noise, were set to 68 dB SPL. These levels were measured at the position of the participant’s head using a sound level meter. During the time-estimation task, this setup yielded a signal-to-noise ratio of −3 dB between the auditory feedback and multi-talker babble noise (Bennett et al., 2012). All the tests were conducted in a soundproof booth to ensure acoustic isolation.

### 2.3 Procedures for measuring SPN

In this study, to enhance the accuracy of measuring the SPN evoked by spoken language stimuli, we employed a time-estimation task that incorporated auditory feedback. The measurement procedures adopted were based on those described by Ohgami et al. (2021) for assessing SPN in response to spoken language stimuli. The participants performed the time-estimation task under two feedback conditions—the speech and speech-in-noise (speech amid multi-talker babble noise) conditions—with pure-tone as the control condition.

**FIGURE 2:**
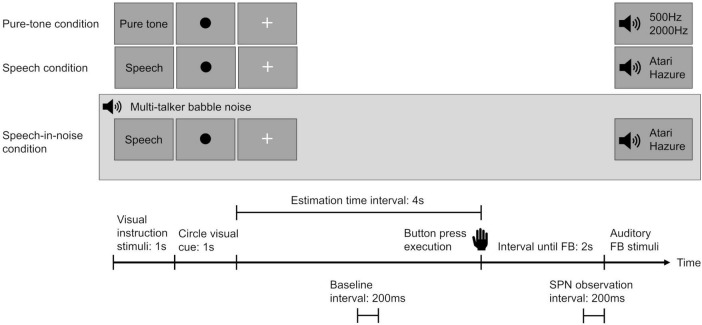
Overview of the experimental design for each condition. Participants were instructed to press a button after estimating the passage of 4 s. Two seconds after pressing the button, an auditory feedback stimulus was presented to convey the accuracy of their time estimation. In the speech-in-noise condition, a background of multi-talker babble noise was introduced. The stimulus-preceding negativity was observed in the interval from –200 ms to 0 ms before the auditory feedback stimulus. The baseline was established using the mean amplitude from 2,000 ms to 1,800 ms seconds before the button press. SPN indicates stimulus-preceding negativity and FB indicates feedback.

During the task, a green circular visual cue was presented on the screen for 1 s, followed by a “+” sign to indicate the trial interval. The participants were instructed to press a button once they perceived that 4 s had passed since the green circular visual cue disappeared. Two seconds after the button was pressed, a feedback stimulus was displayed, informing the participants of their accuracy in time estimation (Figure 2). An accurate range was defined as the interval width for accurate time estimation (e.g., if the defined range was 1 s, the participants were required to press the button at 4 s plus or minus 0.5 s). A preliminary experiment was conducted in each participant to establish an accurate range threshold that yielded a 60% correct response rate for each individual.

Under each condition, two separate auditory feedback stimuli were used to determine the participants’ time-estimation performance accuracy. In both the speech and speech-in-noise conditions, feedback was provided using voices of individuals of the same sex as those of the participants, uttering the Japanese words “Atari” (correct) and “Hazure” (incorrect), to prevent emotional reactions triggered by voices of individuals of the opposite sex. In the pure-tone condition, high (2000 Hz) and low (500 Hz) tones were used. Correctness was represented by the term “Atari” in conjunction with the 2000Hz tone, while the other stimuli indicated incorrectness. A reward of 10 yen (US$ 0.07) was given for precise time estimations to enhance participant concentration and motivation throughout the task.

The participants underwent a training session, followed by three experimental sessions. The training session, comprising a minimum of 12 trials, allowed the participants to familiarize themselves with the task and learn eye movement control, with the number of trials adjusted based on their task comprehension. Each experimental session comprised two consecutive blocks of 30 trials under each condition, resulting in 180 trials (30 trials × 2 blocks × 3 conditions). After each block, participants received feedback on their accuracy percentages and incentive earnings. Each block was followed by a 3-min rest period. The sequence of each condition was randomized. The participants were instructed to use the index finger of their right hand to press buttons and to ensure steady finger movements across all conditions. They were advised against verbal or rhythmic counting during time estimation in order to strive for the highest possible accuracy.

After completing the time-estimation task, participants were asked to self-report their subjective listening effort using a visual analog scale (VAS). This evaluation method was based on previous reports that focused on quantifying subjective listening efforts (Bräcker et al., 2019; Kestens et al., 2023). This study employed a VAS labeled with the words “difficult” and “easy” at opposite ends. A score approaching 0 suggested difficulty in listening, whereas a score nearing 10 indicated ease of listening. This scale was used to evaluate the effort required to perceive auditory feedback during the time-estimation tasks under both the speech and speech-in-noise conditions. The VAS score was measured following each multi-talker babble noise condition block, and the average of the two total measurements was used.

### 2.4 Behavioral auditory processing tests

To assess participants’ speech comprehension performance in noisy environments, we conducted the speech-in-noise test, commonly used in the evaluation of LiD (Bamiou et al., 2001; Bellis, 2003; Geffner and Ross-Swain, 2019). Additionally, we conducted the dichotic listening test to assess individual differences in auditory selective attention, which may influence speech comprehension performance (Broadbent, 1954; Eskicioglu et al., 2019; Obuchi et al., 2023).

#### 2.4.1 Speech-in-noise test

The speech-in-noise test involved the same loudspeaker configuration setup used for SPN measurements to ensure consistency in the auditory conditions. In this test, distinct two-syllable words were used as target stimuli, and the test was designed to minimize the potential influence of attention, working memory, and top-down processing on speech comprehension, thereby focusing on the participants’ auditory perception. The central loudspeaker emitted target stimuli at 65 dB SPL, with the two additional loudspeakers on the left and right delivering multi-talker babble noise at 68 dB SPL. Throughout the test, 36 unique two-syllable words were randomly played through the central loudspeaker, and the participants were instructed to repeat them accurately.

#### 2.4.2 Dichotic listening test

In this test, participants listened to different syllables delivered to their right and left ears through supra-aural headphones (K271, AKG Acoustics, Vienna, Austria) and were asked to accurately repeat the syllables they heard. The task comprised 30 trials, each with two pairs of syllables. Auditory stimuli consisted of six distinct Japanese syllables created from plosive consonants and the vowel “a” (syllables: /pa/, /ta/, /ka/, /ba/, /da/, /ga/). All possible syllable combinations were included in the trials, except for identical pairs. The scoring of responses was based on the accuracy of recalling the stimuli delivered to both the left and right ears.

### 2.5. Physiological recording

Physiological data were collected using an NVX24 EEG amplifier system and MCScap Ag/AgCl electrode set-24 (Medical Computer Systems, Zelenograd, Moscow, Russia). Electrode placement followed the international 10–20 system layout, with 19 typical electrodes (Fp1, Fp2, F3, F4, C3, C4, P3, P4, O1, O2, F7, F8, T3, T4, T5, T6, Fz, Cz, and Pz) used for data acquisition. The central frontopolar site served as the grounding point, and the earlobes (A1 and A2) were used as reference points. Electrode impedances were maintained at < 10 kΩ throughout data collection.

### 2.6 Data analysis

EEG data were recorded at a 500 Hz sampling rate with a 0.5–70 Hz online analog filter. Stimulus delivery was controlled using PsychoPy software (version 2022.1.4; Peirce, 2007). After data collection, the EEG data were pre-processed using EEGLAB (version 2023.0; Delorme and Makeig, 2004). The EEG epochs analyzed ranged from 1,500 ms before button press to 500 ms after auditory feedback. During preprocessing, epochs contaminated by muscle activity artifacts were excluded by first ruling out significant body movements using monitoring via a digital camera. Following this confirmation, only trials with EEG amplitude changes of < 90 μV were selected for further analysis. The threshold for excluding potentials during artifact screening was determined based on the report by Ohgami et al. (2023), in which a time estimation task with auditory feedback was used to measure SPN. Poor-quality data channels were visually identified and corrected using the spline interpolation routine in the EEGLAB processing toolbox. Additionally, an independent component analysis based on the extended Infomax algorithm was performed to remove artifacts caused by eye blinks and movements (Bell and Sejnowski, 1997; Jung et al., 2000).

The analyses included data from participants with at least 20 valid trials under each condition. If any condition did not meet the 20-trial criteria, the entire dataset for the participant was excluded. Baseline correction was performed using the mean amplitude from −2,000 ms to −1,800 ms relative to the button press. This baseline was selected based on methodologies from previous SPN studies that utilized time estimation tasks (Ohgami et al., 2014; Ohgami et al., 2021). The amplitude changes in the negative direction, discernible within 200–0 ms preceding the commencement of feedback stimulation, were defined as the SPN (Raiha et al., 2020). Additionally, we defined the difference in SPN amplitude between the speech in noise condition and the speech condition (calculated by subtracting the SPN amplitude of the speech condition from that of the speech in noise condition) as “the change in SPN amplitude”.

### 2.7 Statistical analysis

First, we investigated whether the auditory feedback conditions, electrodes, or a combination of both influenced the variance in SPN amplitude. Two-way repeated-measures analyses of variance were used to evaluate the main effect and interactions of the three conditions and nine electrodes (frontal [F3, F4, Fz], central [C3, C4, Cz], and parietal [P3, P4, Pz]) on the SPN amplitudes, and Bonferroni method was used for the post hoc tests. The selection of the electrodes for the analysis was based on the study by Ren et al. (2024), in which six electrodes commonly examined in SPN studies were included (F3, F4, C3, C4, P3, P4), along with midline electrodes (Fz, Cz, Pz). We also compared the difference in the self-reported listening effort between the speech and speech-in-noise conditions using the Mann-Whitney U test.

Second, we evaluated the concurrent validity between the SPN amplitude under the two speech feedback conditions (speech and speech-in-noise conditions) and performance in the behavioral auditory processing tests using Pearson’s product-moment correlation coefficients. Previous research has shown that the SPN amplitude increases in the left frontal region before auditory feedback (Brunia and van Boxtel, 2004; Ohgami et al., 2021). From this point onward, we focused our analysis on the SPN amplitude at the F3 electrode. This is because the left frontal region has been shown to influence individual predictive tendencies during speech comprehension tasks in noisy environments (Schubert et al., 2023).

Third, multiple regression analysis was used to investigate the relationship between the change in SPN amplitude induced by background noise and performance in the speech-in-noise test. The dependent variable was the speech-in-noise test performance, while the independent variables included the change in SPN amplitude and performance in the dichotic listening test. Performance in the speech-in-noise test is influenced by auditory selective attention (Haro et al., 2022; Johns et al., 2024) and auditory working memory (Gordon-Salant and Cole, 2016; Lad et al., 2020). Auditory selective attention is a crucial factor in performing the dichotic listening test (Broadbent, 1954; Eskicioglu et al., 2019; Obuchi et al., 2023), and individual differences in auditory working memory are known to significantly influence auditory selective attention in this test (Colflesh and Conway, 2007). Hence, we adjusted for the influence of these variables by including them as independent variables. The adjusted coefficient of multiple determination (adjusted R-square) was used to indicate how much variability in the speech-in-noise test performance was accounted for by the independent variables. Standardized regression coefficients (β) and associated *p* values were determined to assess statistical significance. A stringent *p*-value threshold of < 0.05 was set to ensure that only statistically significant results were considered for interpretation. All data were pre-processed and analyzed using SPSS software (version 28.0.1; IBM, Chicago, USA). A power analysis was also conducted using the G*Power software (version 3.1; Faul et al., 2009).

## 3 Results

**FIGURE 3:**
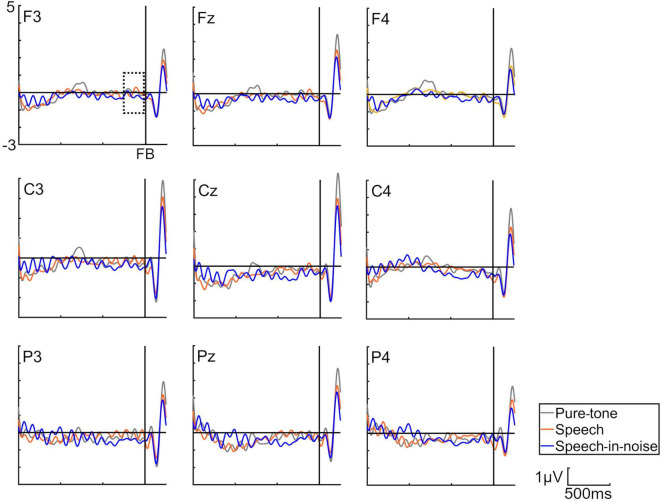
Grand averages of the electroencephalography under the pure-tone, speech, and speech-in-noise conditions. The waveforms in this figure have been filtered (10 Hz low-pass) for visualization purposes only. The delineated dotted square demarcates the temporal window within which stimulus preceding negativity observations were conducted. FB indicate auditory feedback.

**FIGURE 4:**
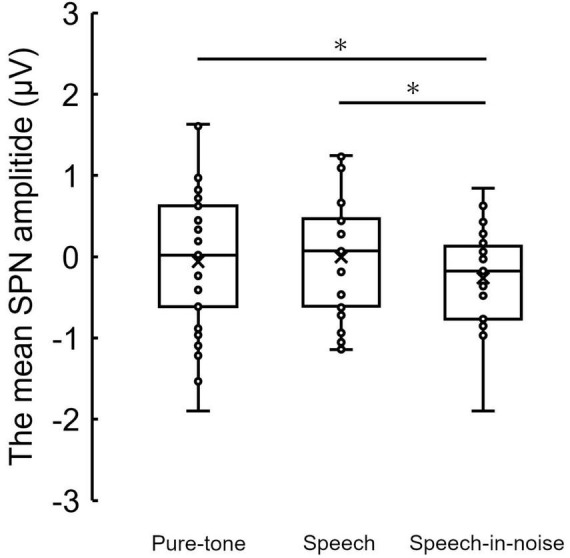
Box plot of the mean SPN amplitude in the pure-tone condition, speech condition, and speech-in-noise condition. *indicate *p* < 0.05. SPN, stimulus-preceding negativity.

**FIGURE 5:**
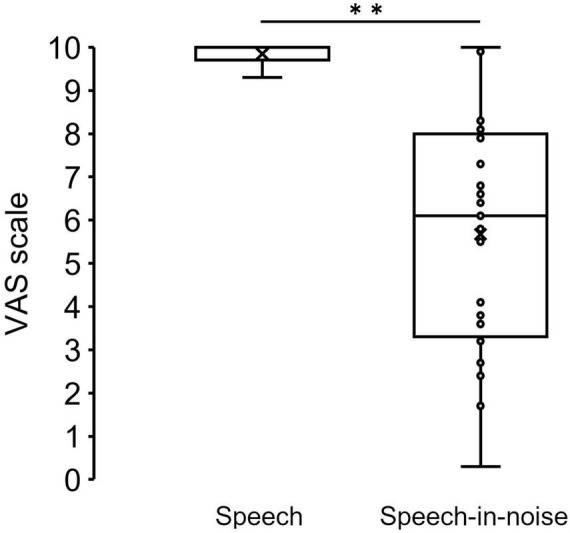
Box plot of the mean VAS scores under the speech and speech-in-noise conditions. **indicate *p* < 0.01. VAS, visual analog scale.

Figure 3 shows the grand average SPN at each electrode. The SPN amplitudes at the bilateral frontal and central electrodes showed a positive peak approximately 600–800 ms after the button press. Following this peak, the amplitudes gradually shifted in a negative direction until the auditory feedback was delivered. By contrast, the SPN amplitudes at the bilateral parietal electrodes showed a negative peak approximately 400–600 ms after the button press, followed by a slight shift in the positive direction until the auditory feedback was delivered. The analysis of the SPN amplitude is summarized in Table 1 and revealed significant main effects of the condition (*F* = 4.06 *p* = 0.018, *f* = 0.39). The results of the Bonferroni corrected post hoc comparisons are shown in Figure 4. SPN amplitude in the speech in noise condition (mean −0.32 μV, SD = 0.63) was significantly higher than in the pure tone condition (mean −0.16 μV, SD = 0.81, *p* = 0.027, *Cohen’s d* = 0.22) and the speech condition (mean −0.17 μV, SD = 0.66, *p* = 0.025, *Cohen’s d* = 0.24). The average self-reported listening effort measured using the VAS was significantly higher under the speech-in-noise condition than under the speech condition (Figure 5: *p* < 0.001). Participants reported greater difficulty in listening under the speech-in-noise condition than under the speech condition.

**TABLE 1:** Results of the analysis of variance pertaining to the mean amplitude of the stimulus-preceding negativity.

Source	*F*	*η* ^2^	*p-*value
Condition	4.06	0.14	0.018
Electrode	0.88	0.03	0.53
Condition × Electrode	0.29	0.006	0.99

**FIGURE 6:**
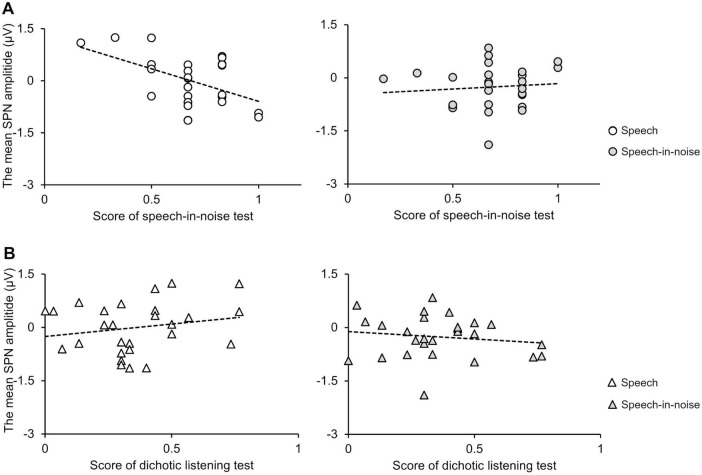
**(A)** Scatter plots showing the correlation between the SPN amplitude at the F3 electrode and speech-in-noise test performance under the speech (left) and speech-in-noise (right) conditions. **(B)** Scatter plots showing the correlation between the SPN amplitude and dichotic listening test performance under the speech (left) and speech-in-noise (right) conditions. SPN, stimulus-preceding negativity.

Under the speech condition, the SPN amplitude at the F3 electrode exhibited a significant negative correlation with performance in the speech-in-noise test (Figure 6A: *r* = −0.49, *p* = 0.009). Note that an increase in the SPN amplitude corresponds to a shift in the negative direction. Under the speech-in-noise condition, the SPN amplitude at the F3 electrode was not correlated with performance in the speech-in-noise test (Figure 6A: *r* = 0.095, *p* = 0.63). This indicates that the influence of prediction on performance in the speech-in-noise test decreases with the addition of multi-talker babble noise. The SPN amplitude at the F3 electrode was not correlated with performance in the dichotic listening test under both the speech (Figure 6B: *r* = 0.21, *p* = 0.32) and speech-in-noise (Figure 6B: *r* = −0.14, *p* = 0.48) conditions.

**FIGURE 7:**
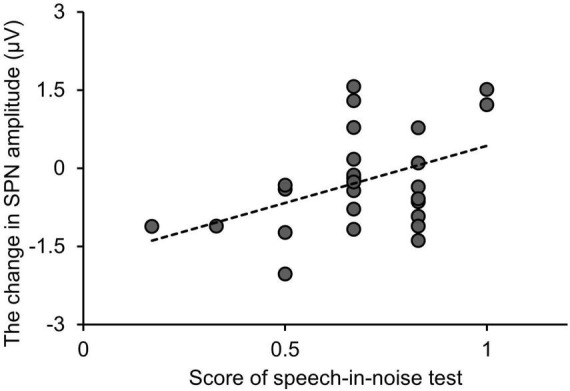
Relationship between changes in the SPN amplitude and performance in the speech-in-noise test. “Change in SPN amplitude” is defined as the SPN amplitude under the speech-in-noise condition minus the SPN amplitude under the speech condition. SPN, stimulus-preceding negativity.

Figure 7 shows the relationship between the SPN amplitude change, i.e., the difference in SPN amplitude between the speech-in-noise and speech conditions, and performance in the speech-in-noise test. Multiple linear regression analysis was conducted to further explore the relationship between the SPN amplitude change and performance in the speech-in-noise test while adjusting for performance in the dichotic listening test. The SPN amplitude change emerged as a significant predictor of performance in the speech-in-noise test (adjusted *R-*square = 0.12, β = 0.017, *p* = 0.038). This predictive relationship remained significant even after adjusting for the potential influences of the performance in the dichotic listening test.

## 4 Discussion

We hypothesized that in noisy environments, the attentional resources required for speech comprehension increase, leading to changes in the SPN amplitude. Additionally, we hypothesized that these changes in the SPN amplitude would be related to performance in behavioral auditory processing tests. These hypotheses were partially confirmed by the following three findings. The SPN amplitude shifted more negatively under the speech-in-noise condition than under the speech and pure-tone conditions. There was a negative correlation between the SPN amplitude under the speech condition and performance in the speech-in-noise test; however, no correlation was observed between the SPN amplitude under the speech-in-noise condition and performance in the speech-in-noise test. Finally, the change in SPN amplitude induced by background noise was related to performance in the speech-in-noise test.

Recent studies on predictive coding have reported that attention increases the gain of neurons, leading to prediction errors (Friston, 2009; Feldman and Friston, 2010). Based on this idea, the increase in the SPN amplitude under the speech-in-noise condition can be interpreted as follows: the addition of background noise decreased the clarity of the input from the bottom-up processing, thereby distorting the auditory feedback loop. To create accurate predictions of speech likely degraded by background noise (note that “accurate” here is used differently from “uncertainty” of the feedback stimulus), more attentional resources are required than those used in the speech condition. This is also suggested by the increased self-reported listening effort in the speech-in-noise condition compared to in the speech condition. Thus, the increase in attentional gain due to the added background noise may be reflected in the SPN amplitude. Similarly, understanding speech in a noisy environment is much more difficult than detecting pure tones in a quiet environment (Rudner and Lunner, 2014). Thus, in the speech-in-noise condition, there might have been an increase in attentional gain and SPN amplitude compared to in the pure-tone condition.

The second finding suggests that prediction is related to performance in speech comprehension tasks in noisy environments. In this experiment, the SPN amplitude under the speech condition was negatively correlated with performance in the speech-in-noise test. However, the SPN amplitude under the speech-in-noise condition was not correlated with performance in the speech-in-noise test. This result partially deviates from our hypothesis. Our findings did not show an association between SPN amplitude and performance in the dichotic listening test. This suggests that SPN amplitude does not reflect the role of selective auditory attention (Kerlin et al., 2010; Lesenfants and Francart, 2020) in challenging speech comprehension tasks with low signal-to-noise ratios, which involve spatially separating competing and target stimuli to focus appropriately on the target stimulus. In other words, the observed correlation between SPN amplitude and performance in the speech-in-noise test may be due to factors other than individual differences in selective auditory attention.

Schubert et al. (2023) reported that individual differences in predictive tendencies observed in the left anterior temporal region are related to performance in speech comprehension tasks in quiet environments. An interesting difference between their findings and ours is that the SPN as an indicator of predictive processes in a quiet environment correlated with performance in speech comprehension tasks in noisy environments. Individuals who exhibit increased SPN amplitude under the speech condition, which is a quiet environment without background noise, may rely more heavily on prediction than on sensory input from bottom-up processing in speech comprehension. Schubert describes this as a “strong predictive tendency.” So far, a strong reliance on prediction has been associated with maladaptive auditory experiences, such as hallucinations and tinnitus (Sedley et al., 2016; Schilling et al., 2023). However, a moderate reliance on prediction may be more robust to distractors, such as background noise, than a strong reliance on sensory input from bottom-up processing. Individuals who moderately rely on prediction in speech comprehension are expected to allocate more attentional resources to generating predictions than those who heavily rely on sensory input from bottom-up processing. This difference in attentional gain may have increased the SPN amplitude. However, it is important to note that the effect size of SPN amplitude under the speech-in-noise condition was relatively small.

The correlation between the SPN amplitude under the speech-in-noise condition and performance in the speech-in-noise test was weaker than that between the SPN amplitude under the speech condition and performance in the speech-in-noise test. This result aligns with those of previous studies showing that the influence of predictive tendencies in the left frontal region changes as the number of distracting speakers increases, making speech comprehension more difficult (Schubert et al., 2023). Together with the third finding, these results indicate individual differences in the degree to which the SPN amplitude increases during speech comprehension tasks in noisy environments. One possible explanation is that there are individual differences in the allocation of the level of attention needed to suppress background noise. The performance of participants who exhibited a greater increase in the SPN amplitude under the speech-in-noise condition than under the speech condition tended to be worse in the speech-in-noise test. The human auditory cortex rapidly and selectively suppresses background noise, enhancing the neural encoding and perception of target stimuli (Leonard et al., 2016; Khalighinejad et al., 2019). Participants who adapted well to background noise likely allocated attention more efficiently, resulting in no significant increase in SPN amplitude. Conversely, participants who struggled to adapt to background noise required more attention, leading to an increase in SPN amplitude. However, this is speculative, as the experimental design did not allow for the differentiation of neural activity related to attention directed at background noise from that related to attention directed at the target stimulus. Additionally, prediction and attention are not mutually exclusive but interactively influence sensory processing (Marzecová et al., 2018). Therefore, further validation studies are needed to confirm these relationships.

Understanding the relationship between SPN and speech comprehension may be beneficial for developing assessment methods for LiD in individuals with normal audiograms. SPN is a predictive neural activity that is not influenced by sensory input from bottom-up processing or the preparation of motor responses. Therefore, unlike traditional behavioral auditory processing tests used to evaluate speech comprehension in LiD, SPN is not affected by peripheral hearing loss undetectable by audiograms. This implies that it may serve as a tool for evaluating the influence of top-down processing in speech comprehension. Additionally, this insight could aid in developing new auditory rehabilitation strategies for individuals struggling with speech perception in challenging auditory environments, offering significant clinical benefits. For example, neuromodulation therapies utilizing SPN (Kukke et al., 2017; Wang and Chen, 2019; Mandalà et al., 2021) could enhance the predictive processes occurring just before speech comprehension, potentially improving the speech comprehension performance.

This study has some limitations. First, the effect size of the SPN amplitude under the speech-in-noise condition was relatively small. This suggests that the practical applications of this result may be limited, and caution is needed when interpreting these findings (Primbs et al., 2023). One possible reason for this could be the impact of the online analog filter. In this study, we applied an online analog filter with a range of 0.5–70 Hz for noise removal. However, the commonly used value for high-pass filters in CNV research, including SPN studies, is 0.05 Hz (Linssen et al., 2011). This difference in filter settings may have affected some or all of the slow negative components of the EEG signals. Second, SPN amplitude is influenced by multiple factors, complicating its interpretation (Kotani et al., 2015; Kotani et al., 2017). For instance, aspects such as arousal state, general emotion, and motivation may affect the modulation of SPN amplitude. Particularly, in the design of this experiment, it is impossible to separate the aspects of motivation related to reward and predictive coding within the SPN components, suggesting that further investigative studies are necessary. Additionally, top-down processing in speech comprehension involve factors, such as language, attention, memory, and working memory (Rudner and Signoret, 2016; Schiller et al., 2022). This study did not investigate which of these factors influenced SPN amplitude in speech comprehension. In conjunction with the first limitation, future research should aim to address these experimental shortcomings and develop protocols to investigate the factors that remain unidentified. Third, the controlled environment necessary for maintaining scientific accuracy may not reflect the complexity and unpredictability of real-world auditory scenarios. While ideal for isolating variables, such a laboratory setting may not consistently represent the varying levels and types of noise encountered in real-life situations. This discrepancy between experimental and real-world conditions could raise concerns about the ecological validity of the conclusions (Protzak and Gramann, 2018).

In conclusion, our results suggest that predictive brain activity, as reflected by SPN amplitude, may serve as a potential indicator of speech comprehension performance in noisy environments. Importantly, SPN represents predictive neural activity that is not influenced by sensory input from bottom-up processing. The application of predictive attentional resources several seconds before a speech perception event may be a factor influencing performance in speech comprehension under noisy conditions. Further validation studies are necessary to confirm the practical applications of these findings.

## Data Availability

The raw data supporting the conclusions of this article will be made available by the authors, without undue reservation.
